# miRNA Profiling of Circulating Small Extracellular Vesicles From Subarachnoid Hemorrhage Rats Using Next-Generation Sequencing

**DOI:** 10.3389/fncel.2020.00242

**Published:** 2020-08-13

**Authors:** Shihai Lan, Lin Zhou, Yimei Wang, Linchun Fang, Le Yang, Suyue Zheng, XinHui Zhou, Bin Tang, Jian Duan, Xiao Wu, Chengxing Yang, Tao Hong

**Affiliations:** ^1^Department of Neurosurgery, The First Affiliated Hospital of Nanchang University, Nanchang, China; ^2^Department of Plastic Surgery, The First Affiliated Hospital of Nanchang University, Nanchang, China

**Keywords:** small extracellular vesicles, plasma, cerebral vasospasm, subarachnoid hemorrhage, miRNA

## Abstract

**Background:**

Extracellular vesicles (EVs) are produced during abnormal and normal physiological conditions. Understanding the expression profile of microRNA (miRNA) in plasma-derived small extracellular vesicles (sEVs) and their roles in subarachnoid hemorrhage (SAH) that cause cerebral vasospasm (CVS) is imperative.

**Methods:**

Sprague Dawley rats (250–300 g) were allocated to sham or SAH groups established using endovascular perforation method. miRNA expression profiles of plasma sEVs in both groups (each *n* = 4) were evaluated using next-generation sequencing (NGS).

**Results:**

There were 142 microRNAs (miRNAs) significantly expressed differently between the two groups, of which 73 were up-regulated while 69 were down-regulated in SAH sEVs compared with those of sham (*p* < 0.05; fold change ≥ 2). The Kyoto Encyclopedia of Genes and Genomes (KEGG) pathway and Gene Ontology (GO) analyses of differently expressed (DE) miRNAs revealed signaling pathways and target genes (TGs) in the SAH group. rno-miR-185-5p, rno-miR-103-3p, rno-miR-15b-3p, rno-miR-93-5p, and rno-miR-98-5p were the top five most up-regulated sEVs miRNAs.

**Conclusion:**

Our results suggest that miRNA can be selectively packaged into sEVs under SAH, and this could help develop potential targets for the prevention, diagnosis, and treatment of CVS after this condition.

## Introduction

Subarachnoid hemorrhage (SAH) is a common hemorrhagic stroke in cerebrovascular accidents, in which aneurysmal SAH (aSAH) accounts for 70–80% of SAH cases (Wong et al., 2014). Cerebral vasospasm (CVS) is a serious complication after aSAH (Dababneh et al., 2014). It occurs 4–7 days after onset of aSAH and peaks in 6–8 days, but relieves after 2 weeks (Kassell et al., 1985; Ingall et al., 2000). The main clinical outcomes for CVS are delayed neurological deficits and delayed cerebral ischemic events, with case fatalities of between 12 and 18% (Eseonu et al., 2016); however, the pathogenesis of this condition remain elusive. Therefore, it is imperative to understand the pathogenesis of CVS, to aid in developing better prevention and treatment interventions.

Exosomes are membrane-bound vesicles (30–150 nm), secreted into the extracellular environment by all types of cells, through the extracellular pathway (Mitchell et al., 2016). Small extracellular vesicles (sEVs) contain a series of lipids, proteins, long non-coding RNAs (lncRNAs), messenger RNAs (mRNAs), and microRNAs (miRNAs). Majority of these vesicles are considered subsets of exosomes (An et al., 2015; Thind and Wilson, 2016). It is interesting to note that miRNAs are transferred via sEVs. miRNAs are stable in this form enabling them to perform their TG regulatory function in recipient cells (Au Yeung et al., 2016).

MicroRNAs on their part are endogenous, 21–23 nt non-coding RNAs modulating gene expression (Etheridge et al., 2011). miRNAs in the brain are important in the formation and functioning of dendritic spines and synaptic plasticity, and normal cognitive function. Since abnormal regulation of miRNA has been associated with many neurological diseases, understanding how they are regulated becomes imperative (Saugstad, 2010). Additionally, miRNAs regulate vascular phenotype by inhibiting or maintaining cell differentiation (Cordes et al., 2009). Individuals with SAH, are at risk of developing several complications such as late cerebral ischemia (LCI) and CVS. Previous studies have shown that gene expression in the cerebral arteries changes significantly after different types of strokes, including SAH, focal cerebral ischemia (FCI), and cardiac arrest (Vikman et al., 2006, 2007; Johansson et al., 2012). Therefore, there is a need to identify biomarkers associated with these complications. miRNAs have been identified in a variety of biological fluids, including plasma, serum, and urine, suggesting that they could be potential minimal invasive biomarkers for certain diseases (Etheridge et al., 2011; Schultz et al., 2014; Vychytilova-Faltejskova et al., 2016).

MicroRNAs in sEVs regulate many physiological and pathological processes (Deng et al., 2017; Figueroa et al., 2017; Teng et al., 2017; Yang et al., 2017; Nguyen et al., 2018). However, their expression profiles in CVS after SAH remain unclear. In this study, we used next-generation sequencing (NGS) to characterize expression profiles of circulating sEVs from sham and SAH group, established using endovascular perforation.

## Materials and Methods

This study conformed with the guidelines on Care and Use of Laboratory Animals of the US National Institutes of Health (NIH publication no. 86-23, revised 1985), while the protocol on the welfare and animal use was approved by the First Affiliated Hospital of Nanchang University.

### Experimental Groups and Induction of Experimental SAH

A total of 100 male rats were obtained from the Department of Laboratory Animal Science, Medical College of Nanchang University, Nanchang, China. At least 1 week before the experiment to the end of the study, rats were reared under controlled conditions of light and temperature, 12/12 h light/dark cycle, 07:00 light on, 24 ± 2°C), with enough food and water. Rats used in this research weighed between 250 and 300 g. The rats were randomly assigned into sham (*n* = 50) or SAH group (*n* = 50). As previous described (Sugawara et al., 2008; Zhang et al., 2018), SAH model (condition) was (engineered) constructed using endovascular perforation method. Briefly, rats were anesthetized with 3% isoflurane in a mixture of oxygen and nitrous oxide (1:2) and operated by sequential operations to expose the internal carotid artery; then, a 4-cm skin incision was made in the ventral neck. Subsequently, we then ligated and transected the external carotid artery after which a blunt 4–0 monofilament nylon thread was advanced into the internal carotid artery about 18 mm. When the operator felt resistance, the nylon thread was advanced a further 3 mm and the wall of the middle cerebral artery (MCA) bifurcation was pierced. After 10 s, the reperfusion through the internal carotid artery was allowed by removal of sutures. For animals in the sham group, similar operations except perforation were performed. After the operation, the rats recovered from their cage for 30–60 min. After recuperating, they were transferred to new clean cages. Seven days after SAH induction, basilar arteries (BAs) for hematoxylin and eosin (H&E) staining were excised after euthanizing.

### H&E Staining and Morphometric Analysis

On the seventh day after SAH, 15 rats/group were killed to obtain specimens for H&E assays. Aestheticization was performed using 3% isoflurane in a mixture of oxygen and nitrous oxide (1:2). We then perfused the heart using 4% paraformaldehyde and 0.1 M phosphate-buffered saline (PBS). The extracted brain tissue was fixed for 2 days with 4% paraformaldehyde, embedded in paraffin, and sectioned into coronal pieces (about 6-μm thick) for H&E staining. The thickness and diameter of BAs at predetermined anatomical locations were measured with ImageJ software package (United States). Stained sections were used to validate successful induction of SAH.

### Blood Collection

Seven days after surgery, four SAH and four sham rats were anesthetized as before, and the abdominal cavity was opened. Eight milliliters of blood was collected from the inferior vena cava in a tube with ethylenediaminetetraacetic acid (EDTA). Plasma was obtained from blood samples by centrifugation for 1 h at 4°C and 3,000 × *g* in a swinging bucket rotor. Plasma was out in a new cone-bottom tube, followed by centrifugation for 15 min at 4°C and 3,000 × *g* to exclude cell debris. Supernatants (clean plasma) were transferred into new tubes, filtered with a 0.22-μm pore filter to exclude larger extracellular vesicles (EVs) and divided equally in small tubes for storage at −80°C.

### Isolation of sEVs

Small extracellular vesicles in the pre-filtered plasma were extracted using the exoEasy Maxi kit, according to the manufacturers’ instructions (Invitrogen, United States, catalog no. 4484450). Briefly, 2 ml of XBP buffer was added to an equal volume of plasma and mixed gently by inverting the tubes five times. The mixture was centrifuged for 1 min in an exoEasy spin column at 500 × *g*. Flow-through was discarded, but the column was put back in the same collection tube, where 10 ml of XWP buffer was added in the column and centrifuged again for 5 min at 3,000 × *g* to remove the remaining (XBP) buffer. The flow-through was discarded with the collection tube, but the spin column was transferred to a new one.

Subsequently, membranes were soaked in 400 μl of XE buffer and incubated for 1 min. This solution was centrifuged at 500 × *g* for 5 min, and the eluate was collected. The eluent was put back on the exoEasy spin column membrane and recollected after another 1 min incubation and 5 min centrifugation at 5,000 × *g*.

### Western Blot Analysis

The sEVs were lysed in a reagent containing a cocktail of protease inhibitors to extract proteins. These proteins were subjected to Western blotting assay using standard procedures. Briefly, after resolution by 10% sodium dodecyl sulfate–polyacrylamide gel electrophoresis (SDS-PAGE), proteins were electrotransferred to a cellulose nitrate membrane (Millipore), which was subsequently incubated at 4°C overnight with either mouse anti-CD63 (1:2000, Abcam), mouse anti-CD9, mouse anti-Tsg101, mouse anti-Alix (all diluted to 1:2,000, Abcam), or mouse anti-Calnexin (1:3,000, Abcam). The complex was then incubated with IRDye 680 secondary antibody, anti-rabbit, or anti-mouse for 1 h at normal laboratory temperature and then characterized by Odyssey infrared imaging system (LI-COR Biosciences).

### Nanoparticle Tracking Analysis

Full sEVs were quantified with the Nano series-Nano-ZS (Malvern) in preparation for dynamic light scattering analysis. The sEVs were slowly injected into the sample pool while avoiding air bubbles. The mixture was then analyzed using standard operating procedure.

### Transmission Electron Microscopy

A 10-μl aliquot of sEVs extracted from rat plasma was put on carbon-coated copper grid and dried. This was followed by negative staining with 2% uranyl acetate. A photomicrograph was obtained with a HITACHI H-7650 transmission electron microscope (HITACHI, Japan).

### RNA Isolation and Construction of RNA Library

Total RNA in plasma-derived sEVs were extracted according to the mirVana miRNA Isolation Kit (Ambion). The RNA integrity was estimated with the Agilent 2100 Bioanalyzer (Agilent Technology, United States), whereas the concentration of RNA was quantified using NanoDrop 2000 (Thermo Fisher Scientific Inc., United States). We constructed small RNA library using 1 μg of total RNA based on the TruSeq Small RNA Sample Prep Kits (Illumina, United States). Briefly, adapters were linked to both ends of total RNA. Complementary DNA (cDNA) was reverse transcribed from this hybrid using PCR. Pieces between 140 and 160 bp were separated from the PCR product and purified to establish a small RNA library. A DNA high-sensitivity chip was used to assess the quality of the library on the Agilent Bioanalyzer 2100 system. Finally, the library was sorted using the Illumina HiSe × Ten platform. In the end, 150 bp paired-end reads were generated. Sequencing and analysis for the small RNA were performed by OE Biotech Co., Ltd. (Shanghai, China).

### Bioinformatic Analysis

Base calls read and converted segments to electronic raw data (also called raw/reads). Low-quality reads containing 5′ primer and poly A tail were filtered to remove these contaminants. Read operation without 3′ adapter, insertion mark, and those <15 or >41 nt were also filtered. At the initial analysis, we determined the length distribution of the clean sequences in the reference genome. Non-coding RNAs were labeled as small nuclear RNAs (snRNAs), transfer RNAs (tRNAs), ribosomal RNAs (rRNAs), etc., Bowtie alignment (Langmead et al., 2009) was done according to Rfam v.10.1^1^ (Griffiths-Jones et al., 2003). miRBase 22 databases^2^ (Griffiths-Jones et al., 2008) were compared to identify and analyze expression patterns of known miRNAs. Mirdeep2 (Friedländer et al., 2012) was used to analyze small annotated RNAs to identify new miRNAs. We then identified the corresponding miRNA star sequence using the miRBase database and the hairpin structure of a pre-miRNA.

Differentially expressed gene (DEG) algorithm (Anders et al., 2012) in the R package was used to calculate the *p* value in the biologically repeated experiments, while Audic-Claverie statistics (Tino, 2009) was used to calculate the *p* value in experiments without biological repetition. Significant differential expression of the miRNAs was done sat *p* < 0.05.

Targets for differently expressed (DE) miRNAs at S ≥ 150, ΔG ≤ −30 kcal/mol, and demand strict 5′ seed pairing were predicted using Miranda software (Enright et al., 2003) Targetfinder on its part (Fahlgren and Carrington, 2010) predicted miRNA targets in plant. The Kyoto Encyclopedia of Genes and Genomes (KEGG) and Gene Ontology (GO) enrichment analyses were conducted for the DE miRNA target genes using hypergeometric R distribution.

### RNA Isolation and RT-PCR

Total RNA was isolated from plasma sEVs of sham and SAH groups using mirVana miRNA Isolation Kit (Ambion). The NanoDrop 2000 spectrophotometer (Thermo Scientific, United States) was employed to assess RNA yield, whereas the RNA integrity was measured using ethidium-bromide-stained agarose gel electrophoresis.

Quantification cDNA was obtained via a two-step reverse transcription PCR (RT-PCR). Ten microliters RT reaction mix consisted of 5 μl of 2 × TS miRNA Reaction Mix, 0.5 μg RNA, and 0.5 μl of TransScrip miRNA RT Enzyme Mix. Reverse transcription was performed at 37°C for 60 min and at 85°C for 5 s to thermally inactivate the transcription. The reaction was controlled in a GeneAmp^®^ PCR system 9700 (Applied Biosystems, United States). We used the nuclease-free water to dilute the RT reaction mixture by 10 times for storage at −20°C.

To synthesize corresponding DNA, 10 μl of the reaction mixture was made up of 1 μl of cDNA, 5 μl of 2 × PerfectStartTM Green qPCR SuperMix, 3.6 μl of nuclease-free water, 0.2 μl of microRNA-specific primer, and 0.2 μl of universal primer using LightCycler^®^ 480 II Real-Time PCR Instrument (Roche, Swiss). The assay was performed in the 384-well optical plate (Roche, Swiss) at an initial 94°C for 30 s, followed by 40 cycles each at 94°C for 5 s, 60°C for 30 s. Each sample was run in triplicate. In the end, the melting curve was analyzed to validate the PCR products. We designed the mRNA-specific primer sequences [using miRBase database (Release 20.0)], but the synthesis was completed by Generay Biotech (Generay, PRC). rno-miR-185-5p: 5′-TGGAGAGAAAGGCAGTTCCTGA-3′; rno-miR-103-3p: Forward: 5′-AGCAGCATTGTACAGGGCTATGA-3′; rno-miR- 15b-3p: Forward: 5′-CGAATCATTATTTGCTGCTCTA-3′; rno- miR-93-5p: Forward: 5′-AAGTGCTGTTCGTGCAGGTAG-3′; rno-miR-98-5p: Forward: 5′-TGAGGTAGTAAGTTGTAT TGTT-3′; 5S: Forward: 5′-GGAGACCGCCTGGGAATA-3′. We employed the standard 2^–ΔΔCt^ method for quantification of target microRNAs expression with 5S rRNA serving as the reference microRNAs.

### Statistical Analysis

GraphPad Prism 5 software was used for data analysis and presentation. Mean differences across groups were analyzed with a two-tailed unpaired *t* test at *p* < 0.05.

## Results

### Mortality Rate and General and Histological Observations of the SAH Model

**FIGURE 1:**
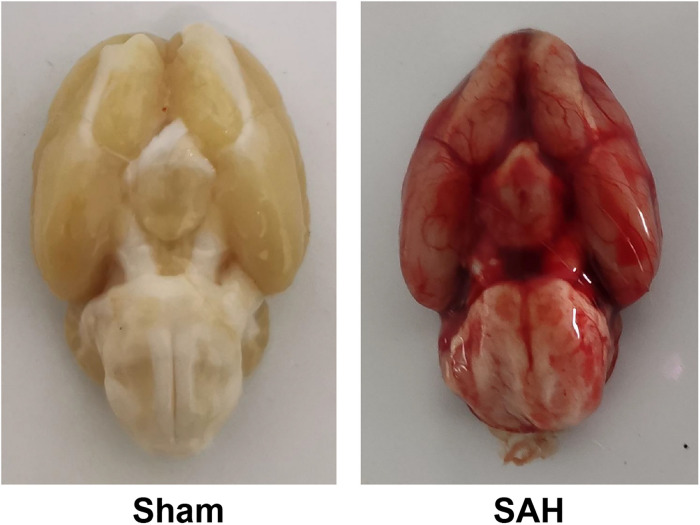
Basal views of the brain in subarachnoid hemorrhage (SAH) and sham groups. Blood clotting was not observed in the sham group, while blood clots are diffused in the subarachnoid space and around Willis’ circle in the SAH group.

**FIGURE 2:**
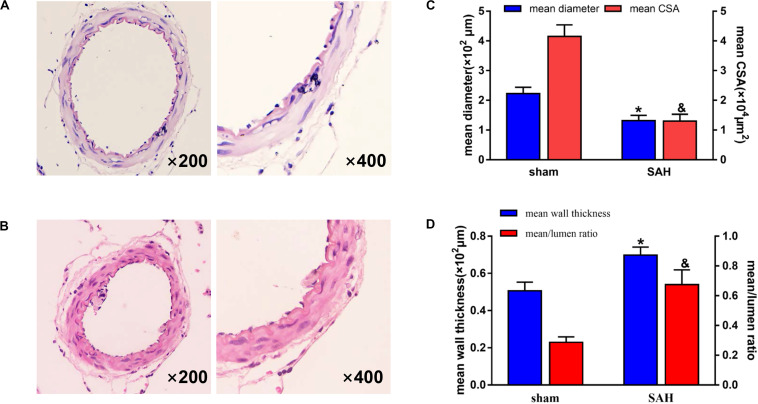
Photomicrographs showing representative histological structural changes of the basilar artery (BA) in each group. H&E staining, magnification, ×200 (left panes) and ×400 (right panes). The group represented in each panel is as follows: **(A)** sham group and **(B)** subarachnoid hemorrhage (SAH) group. There was no vasospasm observed in the sham group. In contrast, morphological vasospasm was observed in the BA rings in the SAH group. **(C)** Quantitative graph of diameter and cross-sectional area (CSA) of the BA in each group. *p < 0.01 vs. sham group in mean diameter, ^&^*p* < 0.01 vs. sham group in mean CSA. *n* = 15. **(D)** Quantitative graph of wall thickness and mean/lumen ratio of the BA in each group. *p < 0.01 vs. sham group in mean wall thickness, ^&^*p* < 0.01 vs. sham group in mean/lumen ratio. *n* = 15. Bars indicate the mean ± SD.

The survival rates in sham and SAH groups were 100% (50/50) and 64% (32/50), respectively, while the overall mortality was 18% (18/100) as presented in Figure 1. Blood clots were seen in the Willis ring and ventral surface of the brainstem in the SAH group. When observed under a light microscope, there were no vasospasms in the sham group. The diameter of BAs and cross-sectional area (CSA) in rats of SAH group were significantly smaller relative to the sham group (Figure 2, *p* < 0.05), the maximal diameter of the sham and SAH groups are 2.619 × 10^2^ and 1.675 × 10^2^ μm, respectively. Additionally, SAH group showed coagulation of chromatin in endothelial cells (ECs), sparse distribution of smooth muscle cells (SMCs), and corrugation of the inner elastic layer. However, no extensive degenerative changes occurred in rats of the sham group.

### Characterization of Plasma-Derived EV-Enriched Fractions

**FIGURE 3:**
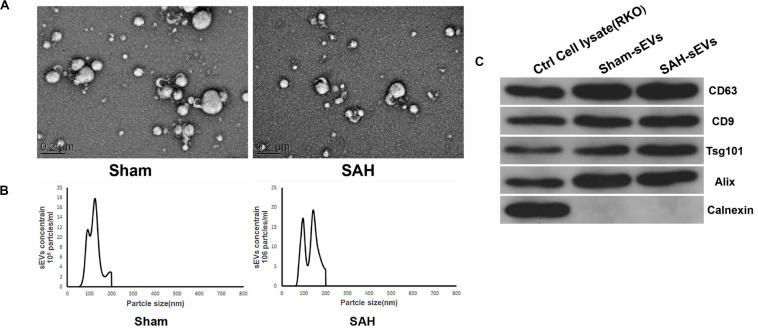
Isolated small extracellular vesicles (sEVs) enriched fractions from sham and subarachnoid hemorrhage (SAH) groups’ plasma. **(A)** Transmission electron microscopy (TEM) images showed that sEVs were oval or bowl-shaped capsules without the nucleus. **(B)** Nanoparticle tracking analysis (NTA) results suggested that sEVs enriched from plasma were about 75–200 nm in diameter. **(C)** sEV markers CD63, CD9, TSG101, and Alix were all detected in the sEV-enriched fractions isolated from the plasma, and Calnexin, a negative marker of sEV was absent in our isolated sEV-enriched fraction samples.

Extraction of sEV-enriched fractions from both groups was performed using the exoEasy Maxi kit, while transmission electron microscopy (TEM) and nanoparticle tracking analysis (NTA) were employed to evaluate the morphology and size distribution of sEVs. The separated sEVs were bowl shaped or oval and ranged from 200 to 75 nm (Figures 3A,B), and they all had CD63, CD9, TSG101, and Alix marker proteins as shown in Figure 3C. In contrast, the negative biomarker of sEVs and Calnexin was missing in the sEV-enriched portion (Figure 3C).

### Differential miRNA Expression Analysis

**FIGURE 4:**
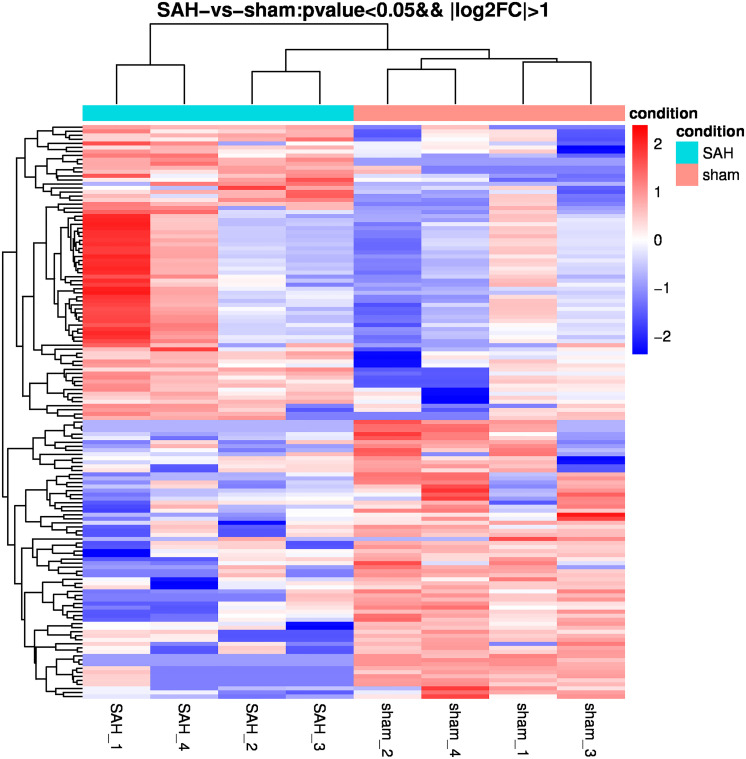
Heatmap of differential microRNA (miRNA) expression between sham and subarachnoid hemorrhage (SAH) small extracellular vesicles (sEVs). Gene expression data were obtained using next-generation sequencing (NGS) on the Illumina HiSeq × Ten platform; the values are presented as reads per kilo base per million mapped reads (RPKM) normalized log2-transformed counts. Red and green colors indicate up- and down-regulated transcripts, respectively.

With a two-fold change, 142 miRNAs in the SAH group were shown to be differentially expressed in sEVs relative to the sham group. Among them, 69 and 73 were down- and up-regulated, respectively. The top 10 differentially expressed miRNAs were rno-miR-130b-3p, rno-miR-872-3p, rno-miR-185-5p, rno-miR-103-3p, rno-miR-19a-3p, rno-let-7d-5p, rno-miR-17-2-3p, rno-let-7i-5p, rno-miR-15b-3p, and rno-miR-362-5p. Figure 4 displays a heat map of DE miRNAs where green and red represent significantly down- and up-regulated miRNAs, respectively.

### KEGG Pathway and GO Enrichment Analyses for the Predicted TGs Associated With the Differentially Expressed miRNAs

**FIGURE 5:**
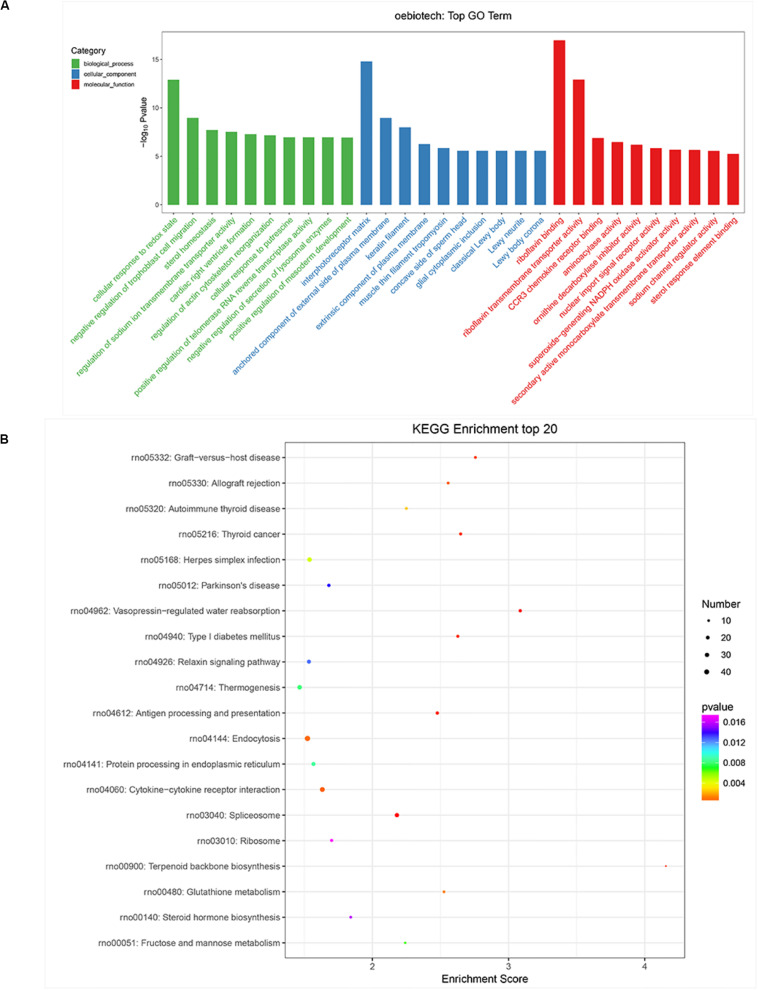
Gene Ontology (GO) enrichment and Kyoto Encyclopedia of Genes and Genomes (KEGG) pathway analysis of differentially expressed genes (DEGs) in subarachnoid hemorrhage (SAH) plasma small extracellular vesicles (sEVs). **(A)** BP, CC, and MF presented the top 10 significance terms of GO enrichment analysis (*p* < 0.05 and FDR < 0.05). **(B)** The top 20 KEGG pathways of significantly DEGs between the control and SAH groups (*p* < 0.05 and FDR < 0.05). GO, Gene Ontology; KEGG, Kyoto Encyclopedia of Genes and Genomes; BP, biological processes; CC, cellular components; MF, molecular functions; FDR, false discovery rate; DE, differentially expressed.

The function of miRNAs can be inferred from the related protein-coding genes (Hata, 2013). We, therefore, explored the potential functions of mRNA-related miRNAs by GO analysis in terms of molecular functions (MFs), cellular components (CCs), and biological processes (BPs). The false discovery rate (*p* < 0.05, ListHits ≧ 3) was set as the selection cutoff value for significance as shown in Figure 5A. Cellular response to redox state was the most enriched term in BPs, while the interphotoreceptor matrix was significant in CCs. In the MF, riboflavin binding was the most enriched as shown in Figure 5A. KEGG pathway analysis revealed that the Relaxin signaling pathway, cytokine–cytokine receptor interaction, endocytosis, thermogenesis, and ribosome (Figure 5B) were within the top 20 enriched. These signaling pathways reflect possible physiological processes during CVS and the potential regulatory mechanisms.

### Validation of Differentially Expressed miRNAs

**FIGURE 6:**
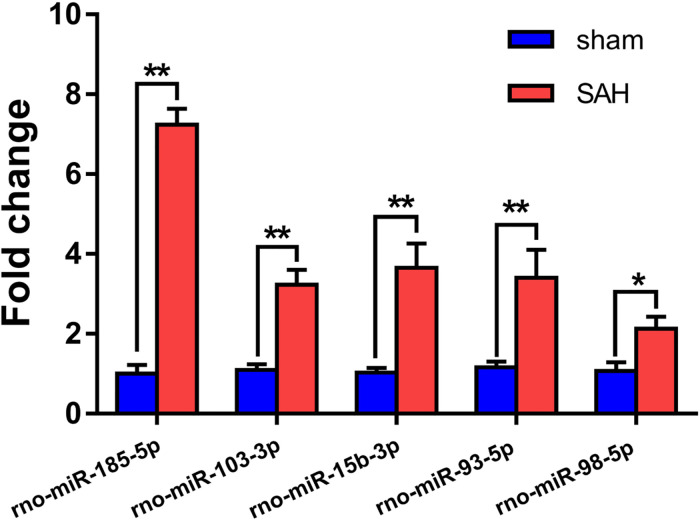
Validation of the differential expression of five miRNAs identified in the microarray using RT-qPCR. The expression levels of plasma small extracellular vesicles (sEVs) miRNA were analyzed in subarachnoid hemorrhage (SAH) (*n* = 10) and sham groups (*n* = 10) using RT-qPCR. A total of five miRNAs were randomly selected, including five up-regulated miRNAs (rno-miR-185-5p, rno-miR-103-3p, rno-miR-15b-3p, rno-miR-93-5p, and rno-miR-98-5p). *p < 0.05 and **p < 0.01. RT-qPCR, reverse transcription quantitative polymerase chain reaction; miR/miRNA, microRNA.

Small extracellular vesicles were isolated from the plasma of 10 rats from the sham and 10 from the SAH groups. Relative expression levels of rno-miR-185-5p, rno-miR-103-3p, rno-miR-15b-3p, rno-miR-93-5p, and rno-miR-98-5p in sEVs from the rats showed that these miRNAs were up-regulated in the SAH group as shown in Figure 6. RT-qPCR of these miRNAs showed similar expression trend as miRNA-sequence data (Table 1).

**TABLE 1:** Top 20 differentially expressed microRNAs (miRNAs).

No.	miRNA_ID	(SAH vs. sham) log2 fold change	*p* value	Up/down
1	rno-miR-211-5p	4.02122	0.0050	Up
2	rno-miR-93-3p	3.6908	0.0152	Up
3	rno-miR-130b-3p	3.2675	0.0046	Up
4	rno-miR-483-3p	3.2283	0.0296	Down
5	rno-miR-212-3p	3.1976	0.0354	Up
6	rno-let-872-3p	2.7171	0.0201	Up
7	rno-miR-185-5p	2.6958	0.0014	Up
8	rno-miR-103-3p	2.6587	0.0087	Up
9	rno-miR-19a-3p	2.4994	0.0320	Up
10	rno-let-7d-5p	2.4710	0.0127	Up
11	rno-miR-17-2-3p	2.4540	0.0337	Up
12	rno-let-7i-5p	2.4408	0.0128	Up
13	rno-miR-15b-3p	2.4287	0.0060	Up
14	rno-miR-362-5p	2.4117	0.0346	Up
15	rno-miR-3559-5p	2.3228	0.0097	Up
16	rno-miR-484	2.3180	0.0126	Up
17	rno-miR-93-5p	2.2430	0.0061	Up
18	rno-miR-98-5p	2.2308	0.0173	Up
19	rno-let-7g-5p	2.2071	0.0173	Up
20	rno-miR-361-3p	2.1984	0.0225	Up

## Discussion

This is the first study investigating circulating sEVs in SAH by small RNA sequencing. We found distinct expression patterns for plasma sEVs miRNA between CVS related to SAH and controls.

Plasma exosomes were isolated using standard methods for the extraction of the sEV-enriched fraction. Theoretically, it is not possible to determine the origin of EVs found in the extracellular environment. Therefore, according to the MISEV2018 Guide (Théry et al., 2018), we employed the term “extracellular vesicle (EV) + specification (size)” instead of “exosomes.” In this study, we held the perspective that the definitions (lEV, sEV) do not accurately reflect the biological meaning but can be experimentally controlled compared to prior definitions and microvesicle/exosome (Mateescu et al., 2017).

Small extracellular vesicles respond to cellular environmental and external stimuli (Burke et al., 2016; Iraci et al., 2016). We postulated that plasma sEV miRNAs are expressed differently in SAH rats compared to sham controls. Thus, we performed NGS on plasma sEVs in the two groups. In total, 142 miRNAs showed significant differential expression. Specifically, 73 and 69 were increased and decreased, respectively. The expression of five randomly selected miRNAs in the two groups was determined using RT-qPCR.

Expression of plasma sEVs rno-miR-185-5p, rno-miR-103-3p, rno-miR-15b-3p, rno-miR-93-5p, and rno-miR-98-5p were most elevated in SAH rats compared with sham group. A recent report (Wang et al., 2017) demonstrated that miR-185/P2Y6 axis might inhibit Ang II-induced human aortic vascular smooth muscle cell (HAVSMC) proliferation via downstream extracellular signal-regulated kinase (ERK) pathway or through negatively regulating P2Y6 expression by miR-185. Additionally, miR-103 has been shown to program ECs toward a maladapted phenotype by targeting lncWDR59, which may promote atherosclerosis (Natarelli et al., 2018). Suppression of miR-103 may accelerate angiogenesis in the context of ischemic stroke and decrease infarct volume by up-regulating vascular endothelial growth factor (VEGF) (Shi et al., 2018). Previously, miR-15b promoted platelet-derived growth factor signaling, thereby increasing the proliferation of vascular smooth muscle cells (Kim and Kang, 2013). Consistently, recent research shows that miR-93 promotes migration and proliferation of VSMCs targeting Mfn2 (Feng et al., 2019). Additionally, let-7g and miR-98 protected the blood–brain barrier in the context of neuroinflammation (Rom et al., 2015). The pathogenesis of CVS is not completely clear, and there is no clear treatment. Its main clinical manifestations are delayed cerebral ischemia (DCI), delayed neurological impairment, and even death, which seriously threaten the life of patients (Lin et al., 2003). Many studies have shown that there is a close relationship between SAH and miRNA in a variety of physiological and pathological aspects, such as signal pathway level, cellular level, and so on, and predict that miRNA will become one of the effective methods for the treatment of SAH in the future (Su et al., 2015; Chen et al., 2017). It not only participates in inflammation, neuronal apoptosis, synaptic remodeling, and many other cellular functions but also detects the differential expression of miRNA in blood, cerebrospinal fluid (CSF) of SAH patients, and MCA of SAH model in rats (Kikkawa et al., 2017; Sheng et al., 2018a, b). Many disorders of miRNA expression are involved in the occurrence and development of CVS, and the detection of miRNA in EVs can be used as a new standard to prevent, diagnose, and judge the prognosis of CVS after SAH. Although the research on the relationship between EV-derived miRNA and CVS is not deep enough, CVS is affected by changing the biological behavior of EV-derived miRNA in the process of SAH, such as changing the expression level of miRNA in EVs, changing miRNA-related genes in EVs, or blocking the action process of EVs.

To study the regulatory roles of miRNAs differentially expressed in sEVs, we predicted and evaluated TGs. Among the TGs, certain miRNAs were shown to be involved in pathways associated with Relaxin signaling pathway, cytokine–cytokine receptor interaction, endocytosis, thermogenesis, and ribosome in SAH. For instance, human relaxin-2 (“relaxin”), originally identified as a peptide hormone during pregnancy, is now known to play a number of multiple roles in both men and women, including vasodilation, antifibrosis, and angiogenesis (Nistri and Bani, 2003; Samuel et al., 2006; Du et al., 2010; Sarwar et al., 2017). Cytokines are soluble extracellular proteins or glycoproteins, which are important regulatory and mobilization factors between cells. They participate in congenital and adaptive inflammatory host defense, cell growth, differentiation, cell death, angiogenesis, and the process of development and repair aimed at restoring homeostasis (Fischer and Hilfiker-Kleiner, 2007; Turner et al., 2014). Therefore, Relaxin signaling pathway and cytokine–cytokine receptor interaction may play an important role in the occurrence and development of CVS.

In view of these, we have reason to believe that CVS after SAH rats is related to the changes in miRNA in plasma. Changes in miRNA may cause changes in the phenotype of blood vessels, resulting in the development of CVS after SAH. As an important regulator, miRNA is closely related to the pathogenesis of SAH. With the development of precision medicine, miRNA is expected to become a cognitive dysfunction disease new target in clinical diagnosis and treatment. sEVs are biological nanoparticles that transmit information between cells and have great potential in the treatment of diseases. One of the most useful properties of sEVs is their ability to cross barriers such as the plasma membrane and blood–brain barrier. This makes them ideal for delivering therapeutic molecules. In the future, sEVs may be able to target SAH patients in the form of miRNAs to reduce the incidence of CVS.

This study has several limitations and will be addressed in a follow-up work. First, a larger sample size needs to be added. In the current study, only a small sample size of four SAH rats and four sham rats was included. Second, quantitative RT-PCR only analyzed a part of miR. Third, this study is only a preliminary exploration in experimental rats, and any clinical relevance of our results needs to be explained in future studies. Although the most common aSAH model is that of SAH, the expression profiles of sEV miRNAs based on other interventions such as two times blood injection in occipital cerebral fossa supplement and studies in human beings under natural cause should be evaluated.

## Conclusion

In conclusion, expression profiles of miRNAs in plasma sEVs under the SAH rat model differ significantly from sham rats. Role, diagnosis, prevention, and treatment potential of circulating sEV miRNAs should be further investigated.

## Data Availability Statement

The authors acknowledge that the data presented in this study must be deposited and made publicly available in an acceptable repository, prior to publication. Frontiers cannot accept a manuscript that does not adhere to our open data policies.

## Ethics Statement

The animal study was reviewed and approved by the Institutional Animal Care and Use Committee of the First Affiliated Hospital of Nanchang University.

## Author Contributions

TH conceived and designed the experiments. SL, LZ, LF, and YW performed the experiments. SL, LZ, and YW analyzed the data. SZ, XZ, and BT contributed to the reagents, materials, and analytical tools. SL wrote the manuscript. LY and TH revised the manuscript. JD, XW, and CY contributed to reference collection and data management. All authors contributed to the article and approved the submitted version.

## Conflict of Interest

The authors declare that the research was conducted in the absence of any commercial or financial relationships that could be construed as a potential conflict of interest.

## Footnotes

1 http://www.sanger.ac.uk/software/Rfam

2 http://www.mirbase.org/

## References

[B1] An T. Qin S. Xu Y. Tang Y. Huang Y. Situ B. (2015). Exosomes serve as tumour markers for personalized diagnostics owing to their important role in cancer metastasis. *J. Extracell Vesicles* 4:27522. 10.3402/jev.v4.27522 26095380 PMC4475684

[B2] Anders S. Reyes A. Huber W. (2012). Detecting differential usage of exons from RNA-seq data. *Genome Res.* 22 2008–2017. 10.1101/gr.133744.111 22722343 PMC3460195

[B3] Au Yeung C. L. Co N. N. Tsuruga T. Yeung T. L. Kwan S. Y. Leung C. S. (2016). Exosomal transfer of stroma-derived miR21 confers paclitaxel resistance in ovarian cancer cells through targeting APAF1. *Nat. Commun.* 7:11150. 10.1038/ncomms11150 PMC4820618 27021436

[B4] Burke J. Kolhe R. Hunter M. Isales C. Hamrick M. Fulzele S. (2016). Stem cell-derived exosomes: a potential alternative therapeutic agent in orthopaedics. *Stem. Cells Int.* 2016:5802529. 10.1155/2016/5802529 PMC4745815 26904130

[B5] Chen Y. Huang L. Wang L. Chen L. Ren W. Zhou W. (2017). Differential expression of microRNAs contributed to the health efficacy of EGCG in in vitro subarachnoid hemorrhage model. *Food Funct.* 8 4675–4683. 10.1039/c7fo01064h 29160895

[B6] Cordes K. R. Sheehy N. T. White M. P. Berry E. C. Morton S. U. Muth A. N. (2009). miR-145 and miR-143 regulate smooth muscle cell fate and plasticity. *Nature* 460 705–710. 10.1038/nature08195 19578358 PMC2769203

[B7] Dababneh H. Guerrero W. Mehta S. Moussavi M. Kirmani J. F. (2014). Possible role of Eptifibatide drip in-patient with aneurysmal subarachnoid hemorrhage in vasospasm prevention. *J. Vasc. Interv. Neurol.* 7 8–13. PMC4188253 25298852

[B8] Deng Z. Rong Y. Teng Y. Zhuang X. Samykutty A. Mu J. (2017). Exosomes miR-126a released from MDSC induced by DOX treatment promotes lung metastasis. *Oncogene* 36 639–651. 10.1038/onc.2016.229 27345402 PMC5419051

[B9] Du X. J. Bathgate R. A. Samuel C. S. Dart A. M. Summers R. J. (2010). Cardiovascular effects of relaxin: from basic science to clinical therapy. *Nat. Rev. Cardiol.* 7 48–58. 10.1038/nrcardio.2009.198 19935741

[B10] Enright A. J. John B. Gaul U. Tuschl T. Sander C. Marks D. S. (2003). MicroRNA targets in *Drosophila*. *Genome Biol.* 5:R1. 10.1186/gb-2003-5-1-r1 PMC395733 14709173

[B11] Eseonu C. I. ReFaey K. Geocadin R. G. Quinones-Hinojosa A. (2016). Postoperative cerebral vasospasm following transsphenoidal pituitary adenoma surgery. *World Neurosurg.* 92 7–14. 10.1016/j.wneu.2016.04.099 27155378

[B12] Etheridge A. Lee I. Hood L. Galas D. Wang K. (2011). Extracellular microRNA: a new source of biomarkers. *Mutat. Res.* 717 85–90. 10.1016/j.mrfmmm.2011.03.004 21402084 PMC3199035

[B13] Fahlgren N. Carrington J. C. (2010). miRNA target prediction in plants. *Methods Mol. Biol.* 592 51–57. 10.1007/978-1-60327-005-2_4 19802588

[B14] Feng S. Gao L. Zhang D. Tian X. Kong L. Shi H. (2019). MiR-93 regulates vascular smooth muscle cell proliferation, and neointimal formation through targeting Mfn2. *Int. J. Biol. Sci.* 15 2615–2626. 10.7150/ijbs.36995 31754334 PMC6854371

[B15] Figueroa J. Phillips L. M. Shahar T. Hossain A. Gumin J. Kim H. (2017). Exosomes from glioma-associated mesenchymal stem cells increase the tumorigenicity of glioma stem-like cells via transfer of miR-1587. *Cancer Res.* 77 5808–5819. 10.1158/0008-5472.can-16-2524 28855213 PMC5668150

[B16] Fischer P. Hilfiker-Kleiner D. (2007). Survival pathways in hypertrophy and heart failure: the gp130-STAT3 axis. *Basic. Res. Cardiol.* 102 279–297. 10.1007/s00395-007-0658-z 17530315

[B17] Friedländer M. R. Mackowiak S. D. Li N. Chen W. Rajewsky N. (2012). miRDeep2 accurately identifies known and hundreds of novel microRNA genes in seven animal clades. *Nucleic Acids Res.* 40 37–52. 10.1093/nar/gkr688 21911355 PMC3245920

[B18] Griffiths-Jones S. Bateman A. Marshall M. Khanna A. Eddy S. R. (2003). Rfam: an RNA family database. *Nucleic Acids Res.* 31 439–441. 10.1093/nar/gkg006 12520045 PMC165453

[B19] Griffiths-Jones S. Saini H. K. van Dongen S. Enright A. J. (2008). miRBase: tools for microRNA genomics. *Nucleic Acids Res.* 36 D154–D158. 17991681 10.1093/nar/gkm952 PMC2238936

[B20] Hata A. (2013). Functions of microRNAs in cardiovascular biology and disease. *Annu. Rev. Physiol.* 75 69–93. 10.1146/annurev-physiol-030212-183737 23157557 PMC5215839

[B21] Ingall T. Asplund K. Mähönen M. Bonita R. (2000). A multinational comparison of subarachnoid hemorrhage epidemiology in the WHO MONICA stroke study. *Stroke* 31 1054–1061. 10.1161/01.str.31.5.1054 10797165

[B22] Iraci N. Leonardi T. Gessler F. Vega B. Pluchino S. (2016). Focus on extracellular vesicles: physiological role and signalling properties of extracellular membrane vesicles. *Int. J. Mol. Sci.* 17:171. 10.3390/ijms17020171 26861302 PMC4783905

[B23] Johansson S. Povlsen G. K. Edvinsson L. (2012). Expressional changes in cerebrovascular receptors after experimental transient forebrain ischemia. *PLoS One* 7:e41852. 10.1371/journal.pone.0041852 22848635 PMC3407123

[B24] Kassell N. F. Sasaki T. Colohan A. R. Nazar G. (1985). Cerebral vasospasm following aneurysmal subarachnoid hemorrhage. *Stroke* 16 562–572. 10.1161/01.str.16.4.562 3895589

[B25] Kikkawa Y. Ogura T. Nakajima H. Ikeda T. Takeda R. Neki H. (2017). Altered expression of MicroRNA-15a and Kruppel-Like Factor 4 in cerebrospinal fluid and plasma after aneurysmal subarachnoid hemorrhage. *World Neurosurg.* 108 909.e3–916.e3. 10.1016/j.wneu.2017.09.008 28893694

[B26] Kim S. Kang H. (2013). miR-15b induced by platelet-derived growth factor signaling is required for vascular smooth muscle cell proliferation. *BMB Rep.* 46 550–554. 10.5483/bmbrep.2013.46.11.057 24152911 PMC4133843

[B27] Langmead B. Trapnell C. Pop M. Salzberg S. L. (2009). Ultrafast and memory-efficient alignment of short DNA sequences to the human genome. *Genome Biol.* 10:R25. 10.1186/gb-2009-10-3-r25 PMC2690996 19261174

[B28] Lin C. L. Calisaneller T. Ukita N. Dumont A. S. Kassell N. F. Lee K. S. (2003). A murine model of subarachnoid hemorrhage-induced cerebral vasospasm. *J. Neurosci. Methods* 123 89–97. 10.1016/s0165-0270(02)00344-8 12581852

[B29] Mateescu B. Kowal E. J. van Balkom B. W. Bartel S. Bhattacharyya S. N. Buzás E. I. (2017). Obstacles and opportunities in the functional analysis of extracellular vesicle RNA - an ISEV position paper. *J. Extracell Vesicles* 6:1286095. 10.1080/20013078.2017.1286095 28326170 PMC5345583

[B30] Mitchell M. D. Scholz-Romero K. Reed S. Peiris H. N. Koh Y. Q. Meier S. (2016). Plasma exosome profiles from dairy cows with divergent fertility phenotypes. *J. Dairy Sci.* 99 7590–7601. 10.3168/jds.2016-11060 27372594

[B31] Natarelli L. Geißler C. Csaba G. Wei Y. Zhu M. di Francesco A. (2018). miR-103 promotes endothelial maladaptation by targeting lncWDR59. *Nat. Commun.* 9:2645. 10.1038/s41467-018-05065-z PMC6035258 29980665

[B32] Nguyen M. A. Karunakaran D. Geoffrion M. Cheng H. S. Tandoc K. Perisic Matic L. (2018). Extracellular vesicles secreted by atherogenic macrophages transfer MicroRNA to inhibit cell migration. *Arterioscler. Thromb. Vasc. Biol.* 38 49–63. 10.1161/atvbaha.117.309795 28882869 PMC5884694

[B33] Nistri S. Bani D. (2003). Relaxin receptors and nitric oxide synthases: search for the missing link. *Reprod. Biol. Endocrinol.* 1:5. 10.1186/1477-7827-1-5 12646076 PMC151800

[B34] Rom S. Dykstra H. Zuluaga-Ramirez V. Reichenbach N. L. Persidsky Y. (2015). miR-98 and let-7g^∗^ protect the blood-brain barrier under neuroinflammatory conditions. *J. Cereb. Blood Flow Metab.* 35 1957–1965. 10.1038/jcbfm.2015.154 26126865 PMC4671116

[B35] Samuel C. S. Du X. J. Bathgate R. A. Summers R. J. (2006). ‘Relaxin’ the stiffened heart and arteries: the therapeutic potential for relaxin in the treatment of cardiovascular disease. *Pharmacol. Ther.* 112 529–552. 10.1016/j.pharmthera.2005.05.012 16814863

[B36] Sarwar M. Du X. J. Dschietzig T. B. Summers R. J. (2017). The actions of relaxin on the human cardiovascular system. *Br. J. Pharmacol.* 174 933–949. 10.1111/bph.13523 27239943 PMC5406304

[B37] Saugstad J. A. (2010). MicroRNAs as effectors of brain function with roles in ischemia and injury, neuroprotection, and neurodegeneration. *J. Cereb. Blood Flow Metab.* 30 1564–1576. 10.1038/jcbfm.2010.101 20606686 PMC2932764

[B38] Schultz N. A. Dehlendorff C. Jensen B. V. Bjerregaard J. K. Nielsen K. R. Bojesen S. E. (2014). MicroRNA biomarkers in whole blood for detection of pancreatic cancer. *JAMA* 311 392–404. 24449318 10.1001/jama.2013.284664

[B39] Sheng B. Fang X. Liu C. Wu D. Xia D. Xu S. (2018a). Persistent High Levels of miR-502-5p Are associated with poor neurologic outcome in patients with aneurysmal subarachnoid hemorrhage. *World Neurosurg.* 116 e92–e92. 10.1016/j.wneu.2018.04.088 29689401

[B40] Sheng B. Lai N. S. Yao Y. Dong J. Li Z. B. Zhao X. T. (2018b). Early serum miR-1297 is an indicator of poor neurological outcome in patients with aSAH. *Biosci. Rep.* 38:BSR20180646. 10.1042/BSR20180646 30355655 PMC6246762

[B41] Shi F. P. Wang X. H. Zhang H. X. Shang M. M. Liu X. X. Sun H. M. (2018). MiR-103 regulates the angiogenesis of ischemic stroke rats by targeting vascular endothelial growth factor (VEGF). *Iran J. Basic Med. Sci.* 21 318–324. 29511499 10.22038/IJBMS.2018.27267.6657 PMC5817176

[B42] Su X. W. Chan A. H. Lu G. Lin M. Sze J. Zhou J. Y. (2015). Circulating microRNA 132-3p and 324-3p profiles in patients after acute aneurysmal subarachnoid hemorrhage. *PLoS One* 10:e0144724. 10.1371/journal.pone.0144724 26675167 PMC4682983

[B43] Sugawara T. Ayer R. Jadhav V. Zhang J. H. (2008). A new grading system evaluating bleeding scale in filament perforation subarachnoid hemorrhage rat model. *J. Neurosci. Methods* 167 327–334. 10.1016/j.jneumeth.2007.08.004 17870179 PMC2259391

[B44] Teng Y. Ren Y. Hu X. Mu J. Samykutty A. Zhuang X. (2017). MVP-mediated exosomal sorting of miR-193a promotes colon cancer progression. *Nat. Commun.* 8:14448. 10.1038/ncomms14448 PMC5321731 28211508

[B45] Théry C. Witwer K. W. Aikawa E. Alcaraz M. J. Anderson J. D. Andriantsitohaina R. (2018). Minimal information for studies of extracellular vesicles 2018 (MISEV2018). a position statement of the International Society for Extracellular Vesicles and update of the MISEV2014 guidelines. *J. Extracell Vesicles* 7:1535750. 10.1080/20013078.2018.1535750 PMC6322352 30637094

[B46] Thind A. Wilson C. (2016). Exosomal miRNAs as cancer biomarkers and therapeutic targets. *J. Extracell Vesicles* 5:31292. 10.3402/jev.v5.31292 27440105 PMC4954869

[B47] Tino P. (2009). Basic properties and information theory of Audic-Claverie statistic for analyzing cDNA arrays. *BMC Bioinformatics* 10:310. 10.1186/1471-2105-10-310 19775462 PMC2761412

[B48] Turner M. D. Nedjai B. Hurst T. Pennington D. J. (2014). Cytokines and chemokines: at the crossroads of cell signalling and inflammatory disease. *Biochim. Biophys. Acta* 1843 2563–2582. 10.1016/j.bbamcr.2014.05.014 24892271

[B49] Vikman P. Ansar S. Henriksson M. Stenman E. Edvinsson L. (2007). Cerebral ischemia induces transcription of inflammatory and extracellular-matrix-related genes in rat cerebral arteries. *Exp. Brain Res.* 183 499–510. 10.1007/s00221-007-1062-5 17828393

[B50] Vikman P. Beg S. Khurana T. S. Hansen-Schwartz J. Edvinsson L. (2006). Gene expression and molecular changes in cerebral arteries following subarachnoid hemorrhage in the rat. *J. Neurosurg.* 105 438–444. 10.3171/jns.2006.105.3.438 16961140

[B51] Vychytilova-Faltejskova P. Radova L. Sachlova M. Kosarova Z. Slaba K. Fabian P. (2016). Serum-based microRNA signatures in early diagnosis and prognosis prediction of colon cancer. *Carcinogenesis* 37 941–950. 10.1093/carcin/bgw078 27485599

[B52] Wang S. Tang L. Zhou Q. Lu D. Duan W. Chen C. (2017). miR-185/P2Y6 axis inhibits angiotensin ii-induced human aortic vascular smooth muscle cell proliferation. *DNA Cell Biol.* 36 377–385. 10.1089/dna.2016.3605 28277742

[B53] Wong G. K. Wun Tam Y. Y. Zhu X. L. Poon W. S. (2014). Incidence and mortality of spontaneous subarachnoid hemorrhage in Hong Kong from 2002 to 2010: a Hong Kong hospital authority clinical management system database analysis. *World Neurosurg.* 81 552–556. 10.1016/j.wneu.2013.07.128 24067740

[B54] Yang V. K. Loughran K. A. Meola D. M. Juhr C. M. Thane K. E. Davis A. M. (2017). Circulating exosome microRNA associated with heart failure secondary to myxomatous mitral valve disease in a naturally occurring canine model. *J. Extracell Vesicles* 6:1350088. 10.1080/20013078.2017.1350088 28804599 PMC5533140

[B55] Zhang Z. Liu J. Fan C. Mao L. Xie R. Wang S. (2018). The GluN1/GluN2B NMDA receptor and metabotropic glutamate receptor 1 negative allosteric modulator has enhanced neuroprotection in a rat subarachnoid hemorrhage model. *Exp. Neurol.* 301 13–25. 10.1016/j.expneurol.2017.12.005 29258835

